# Quercetin alleviates diastolic dysfunction and suppresses adverse pro-hypertrophic signaling in diabetic rats

**DOI:** 10.3389/fendo.2022.1029750

**Published:** 2022-12-08

**Authors:** Linda Bartosova, Csaba Horvath, Peter Galis, Kristina Ferenczyova, Barbora Kalocayova, Adrian Szobi, Adriana Duris-Adameova, Monika Bartekova, Tomas Rajtik

**Affiliations:** ^1^ Department of Pharmacology and Toxicology, Faculty of Pharmacy, Comenius University, Bratislava, Slovakia; ^2^ Institute for Heart Research, Centre of Experimental Medicine, Slovak Academy of Sciences, Bratislava, Slovakia; ^3^ Institute of Physiology, Faculty of Medicine, Comenius University, Bratislava, Slovakia

**Keywords:** quercetin, diabetes, diastolic dysfunction, hypertrophy, remodeling

## Abstract

**Introduction:**

Quercetin (Que) is a potent anti-inflammatory and antioxidant flavonoid with cardioprotective potential. However, very little is known about the signaling pathways and gene regulatory proteins Que may interfere with, especially in diabetic cardiomyopathy. Therefore, we aimed to study the potential cardioprotective effects of Que on the cardiac phenotype of type 2 diabetes mellitus (T2DM) accompanied by obesity.

**Methods:**

For this experiment, we used Zucker Diabetic Fatty rats (fa/fa) and their age-matched lean controls (fa/+) that were treated with either vehicle or 20 mg/kg/day of Que for 6 weeks. Animals underwent echocardiographic (echo) examination before the first administration of Que and after 6 weeks.

**Results:**

After the initial echo examination, the diabetic rats showed increased E/A ratio, a marker of left ventricular (LV) diastolic dysfunction, in comparison to the control group which was selectively reversed by Que. Following the echo analysis, Que reduced LV wall thickness and exhibited an opposite effect on LV luminal area. In support of these results, the total collagen content measured by hydroxyproline assay was decreased in the LVs of diabetic rats treated with Que. The follow-up immunoblot analysis of proteins conveying cardiac remodeling pathways revealed that Que was able to interfere with cardiac pro-hypertrophic signaling. In fact, Que reduced relative protein expression of pro-hypertrophic transcriptional factor MEF2 and its counter-regulator HDAC4 along with pSer^246^-HDAC4. Furthermore, Que showed potency to decrease GATA4 transcription factor, NFAT3 and calcineurin, as well as upstream extracellular signal-regulated kinase Erk5 which orchestrates several pro-hypertrophic pathways.

**Discussion:**

In summary, we showed for the first time that Que ameliorated pro-hypertrophic signaling on the level of epigenetic regulation and targeted specific upstream pathways which provoked inhibition of pro-hypertrophic signals in ZDF rats. Moreover, Que mitigated T2DM and obesity-induced diastolic dysfunction, therefore, might represent an interesting target for future research on novel cardioprotective agents.

## Introduction

The pathological phenotype underlying type 2 diabetes mellitus (T2DM) represents a major risk factor for the development of concurrent cardiac pathologies (diabetic cardiomyopathy). Even though the early stages of diabetic cardiomyopathy might be asymptomatic (1), the early left ventricular (LV) diastolic dysfunction (LVDD) has (LVDD) has been identified as a recurring sign of diabetic myocardium (2). The highly prevalent feature that is associated with diabetic cardiomyopathy in T2DM patients, especially in the presence of metabolic disease, is left ventricular hypertrophy (LVH). Independently of present hypertension, obesity and concomitant T2DM are major triggering factors for the development of LVH (3). It was previously confirmed by multiple studies that hyperglycemia plays a role in the induction of heart remodeling, i.e., cardiac fibroblast proliferation, collagen accumulation and hypertrophic wall enlargement (4, 5). In general, hypertrophy is primarily activated as an adaptation mechanism but can later progress to maladaptive changes resulting in ventricle dilatation and even heart failure. During hypertrophic growth, the fetal gene expression program (e.g., expression of atrial natriuretic peptide or B-type natriuretic peptide) is re-activated which involves several transcription factors such as myocyte enhancer factor-2 (MEF2), nuclear factor of activated T cells (NFAT), GATA and/or serum response factor (SRF) (6). Multiple lines of evidence implicated MEF2 in the pro-hypertrophic and pro-fibrotic cardiac growth (7–10); however, its role, as well as its associated pathways in diabetic myocardium require more detailed analysis. MEF2 activation was reported to be enhanced in diabetic hypertrophy (10), promoting transcription of cardiac structural genes what results in the production of new contractile proteins (11). The induction of MEF2 can be mediated by Ca^2+^-dependent pathways which include the calcium/calmodulin-dependent protein kinase II (CaMKII)/histone deacetylase 4 (HDAC4) signaling network. Class II histone deacetylases (HDACs) are responsible for the epigenetic regulation and direct repression of MEF2 and serve as a substrate for CaMKII (12, 13). When phosphorylated, HDAC4 is exported out of the nucleus and dissociated from MEF2, thereby allowing it to function as an active transcriptional factor (14). Class II HDACs were similarly linked to the repression of other hypertrophy-encompassing factors – NFAT, GATA or SRF (14). Calcineurin on the other hand dephosphorylates NFAT thereby promoting its nuclear import and gene transcription activity (15). The regulatory assembly of cardiac hypertrophy further employs zinc finger-containing transcription factor GATA4 which is a crucial component implicated in hypertrophic growth and heart development (16) whose elevated levels were also associated with diabetes (17–19). Either directly or indirectly, the signal transduction machinery is further mediated by a group of mitogen-activated protein kinases (MAPKs), such as extracellular signal-regulated kinase (Erk)1/2, also known as p44/p42 MAPK and atypical transcriptionally active MAPK Erk5 that respond to a variety of extracellular stimuli associated with hypertrophic program (13, 20).

Despite all the evidence, the mechanism by which insulin resistance or hyperglycemia promote cardiac remodeling remains insufficiently described and therapeutic approaches capable of treating diabetic cardiomyopathy are limited. For this purpose, we aimed to advance the current understanding of previously depicted cardioprotective and anti-remodeling effects of Que which is a natural bioactive polyphenolic compound that belongs to the flavonoid family and is recognized mostly for its strong anti-inflammatory and antioxidative properties (21). Being linked to several documented cardioprotective effects (22) in the models of myocardial ischemia/reperfusion injury (23–25), myocardial infarction (26) or cardiac hypertrophy (27, 28), the exact molecular and protein targets of Que as well as its role in more complex or combined diseases, such as very common coexisting condition – obesity accompanied by T2DM, remain elusive.

## Materials and methods

### Experimental animals and study design

The study design involves rats harboring a missense mutation in the leptin receptor gene which was previously established as a model of T2DM associated with obesity (29). Adult male 1-year-old Zucker Diabetic Fatty (ZDF) rats (fa/fa) and their lean controls (fa/+) (Dobra Voda, Slovak Republic) were consequently divided into four experimental groups – control (fa/+) vehicle-treated group (C, n = 12), diabetic (fa/fa) vehicle-treated group (DIA, n = 16), control (fa/+) Que-treated group (CQ, n = 13) and diabetic (fa/fa) Que-treated group (DQ, n = 17). All rats were fed with normal chow KZ-P/M (complete feed mixture for rats and mouse, reg. no 6147, Dobra Voda, Slovak Republic) and had access to drinking water *ad libitum* in an environment with a stable temperature of 22 ± 2°C and humidity of 45–65%. Quercetin (Sigma Aldrich, cat. No Q4951, St. Louis, MO, USA) at a dose of 20 mg/kg/day was dissolved in a small volume of ethanol and administered on a piece of biscuit (vehicle), previously described by Ferenczyova et al. (29), to Que-treated control (CQ) and diabetic (DQ) group during 6 week-long experimental protocol. Various biochemical (e.g. plasma glucose levels) and metabolic parameters (e.g. lipid profile) have been already presented elsewhere (29–31). During the course of the study protocol, all experiments concerning animals were performed accordingly to the rules issued by the State Veterinary Administration of the Slovak Republic, legislation No 377/2012 and with the regulations of the Animal Research and Care Committee of the Centre of Experimental Medicine SAS—Project no. 2237/18-221/3, approved on 21 of August 2018.

### Echocardiography

Transthoracic echocardiography (echo) was performed at two different time points (before the onset of the treatment (week 0) and at the end of the treatment course (week 6)) using an ultrasound machine GE Healthcare Vivid E9 (GE Healthcare, USA) with a 15.0-MHz transducer probe and ultrasound gel to enhance imaging. The numbers of animals per group subjected to the examination were as follows: CQ, n = 8; DQ, n = 15. Prior to the examination itself, rats were anaesthetized with a continuous supply of isoflurane (Forane, Abbive, USA) mixed with oxygen in the anesthesia induction chamber. Parasternal long axis (PLAX), short axis (SAX) and apical four chambers (A4C) viewing methods were applied in order to obtain left ventricular functional and structural parameters *via* the two-dimensional mode (2-D), Motion-mode (MM), Color Doppler and Pulse wave Doppler. Data accuracy was ensured by performing the exam in a tightly controlled time frame. EchoPac software (GE Healthcare, USA) was used to analyze and then extract the echocardiographic parameters from the captured images. As a marker of diastolic function, we determined the ratio of peak velocity blood flows from early (E) to late (A) diastolic mitral inflow - E/A ratio. Systolic function was assessed through the parameters of cardiac index (CI) (calculated as the cardiac output divided by body weight), heart rate (HR), fractional shortening (FS), end-diastolic volume (EDV) and end-systolic volume (ESV). The left ventricular structure was characterized by interventricular septal thickness in end-diastole (IVSd), left ventricular posterior wall thickness in end-diastole (LVPWd), left ventricular internal diameter in end-diastole (LVIDd) and relative wall thickness (RWT).

### SDS-PAGE and immunoblotting

Left ventricular tissue samples (C, n = 6; CQ, n = 6; D, n = 8; DQ, n = 8) from experimental animals were processed into whole cell lysates by previously established protocol (32) and electrophoretically separated and transferred onto PVDF (polyvinylidene difluoride) membrane. These membranes were incubated with primary antibodies against CaMKIIδ (ab181052, Abcam, UK), Erk5 (#12950, Cell Signaling, USA), GATA-4 (#36966, Cell Signaling, USA), HDAC4 (#5392, Cell Signaling, USA), MEF2A + MEF2C (ab197070, Abcam, UK), NFAT3 (#2183, Cell Signaling, USA), pan-Akt (#4691, Cell Signaling, USA), Pan-Calcineurin A (#2614, Cell Signaling Technology, USA), p44/42 MAPK (Erk1/2) (#4695, Cell Signaling, USA), Phospho-HDAC4 (Ser246)/HDAC5 (Ser259)/HDAC7 (Ser155) (#3443, Cell Signaling, USA), phospho-p44/42MAPK (Erk1/2) (Thr202/Tyr204) (#4377, Cell Signaling, USA), phospho-T286-CaMKII (ab171095, Abcam, UK), PPP1CB (ab53315, Abcam, UK), PP2A C Subunit (#2259, Cell Signaling, USA), Phospho-Akt (Thr308) (#4056, Cell Signaling, USA), proBNP (ab239514, Abcam, UK), SRF (ab53147, Abcam, UK). Following the incubation with primary antibodies, these membranes were incubated with HRP-conjugated secondary antibodies: donkey anti‐rabbit IgG (711-035-152, Jackson Immunoresearch), donkey anti‐rat IgG (112-035-175, Jackson Immunoresearch, USA) and donkey anti‐mouse IgG (115-035-174, Jackson Immunoresearch, USA). To detect and capture protein signals, we used enhanced chemiluminescence (Crescendo Luminata, Merck Millipore, USA) and a chemiluminescence imaging system (myECL imager, Thermo Scientific, USA). The quantification of relative protein expression was performed by normalizing the protein band intensity with the intensity of its whole lane protein. As a loading control, we used total protein staining of the membrane with Ponceau S assessed by scanning densitometry. For scanning densitometry, My Image Analysis software (Thermo Scientific, USA) was used.

### Hydroxyproline assay

The left ventricular collagen content was determined by a hydroxyproline assay described previously (33). Numbers of animals per group subjected to the examination were as follows: C, n = 6; CQ, n = 7; DIA, n = 8; DQ, n = 9. Briefly, homogenized LV tissue samples were incubated with NaOH at 115°C to hydrolyze proteins, excessive NaOH was later on neutralized in cooled samples with H_2_SO_4_. Samples were subsequently centrifugated and the supernatant was collected. According to the protocol, samples were first incubated with Chloramine-T reagent and secondly with DMAB (p‐dimethylaminobenzaldehyde) reagent and hydroxyproline concentration was measured spectrophotometrically at 560 nm. Final collagen content, which is presented as a percentage of wet tissue weight, was calculated by dividing the hydroxyproline concentration by a factor of 0.135.

### Statistical analysis

All the presented results underwent statistical analysis using GraphPad Prism7 (GraphPad Software, USA). The data obtained from echo recordings at two different time points (before the Que administration and after the treatment course) were analyzed by two-way ANOVA with Holm-Sidak´s multiple comparisons test. For the protein expression analysis and collagen content determination, we used one-way ANOVA and Tukey´s *post hoc* test. All data are presented as mean ± standard error of the mean (SEM). The power analysis was applied to calculate the sample size with the use of previously obtained experimental data and the significance level of α = 0.05 and power of 1-β = 0.8.

## Results

### Quercetin selectively improved diastolic dysfunction through modulation of the E/A ratio

**Figure 1:**
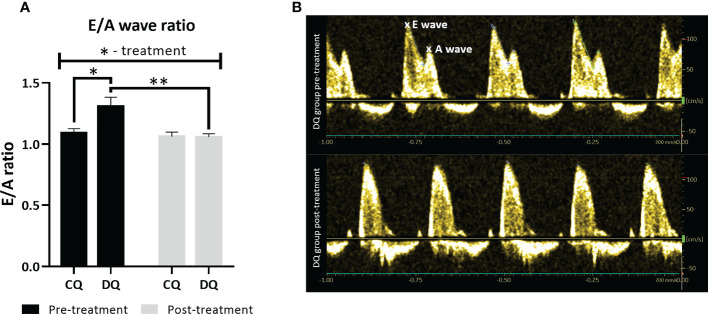
The assessment of LV diastolic function at two different time points – pre-treatment (week 0 – prior to the administration of Que) and post-treatment (week 6 – at the end of the Que treatment course). **(A)** E/A ratio of transmitral inflow from early (E wave) to late (A wave) diastole. **(B)** Representative echocardiographic image of E/A ratio in DQ group pre-treatment vs. post-treatment. Data are presented as mean ± SEM and statistical differences as *p < 0.05, **p < 0.01 (two-way ANOVA paired with Holm-Sidak´s multiple comparisons test).

In the early echo analysis, prior to Que treatment, we found that diabetic ZDF rats exhibited a significant increase in E/A ratio (**Figure 1A**, DQ pre-treatment vs. CQ pre-treatment *p* < 0.05), a ratio of transmitral inflow between early to late diastole peak velocity blood flow, which is widely accepted to be one of the markers of LVDD. After 6 weeks of Que treatment, the E/A ratio of diabetic rats (DQ post-treatment) improved significantly and was reduced to the level of control lean animals (**Figure 1A**, DQ post-treatment vs. DQ pre-treatment *p* < 0.01). Regarding the therapeutic potential of Que, this is an indicator of its ability to target and alleviate LVDD selectively in diseased diabetic rats. Recorded data of systolic function are presented in **Table 1**. Diabetic animals had decreased heart rate compared to lean animals, although these parameters remained unaffected by treatment (group factor *p* < 0.01). CI, calculated as, calculated as the cardiac output divided by body weight, remained stable throughout the experiment, nevertheless we detected a diabetes-independent on effect of Que on FS which tended to decrease (treatment factor *p* < 0.05) in treated animals. LV filling volumes – EDV and ESV were increased in diabetic animals (**Table 1**, EDV: group factor *p* < 0.001 and DQ pre-treatment vs. CQ pre-treatment *p* < 0.05; ESV: group factor *p* < 0.05) and were further enhanced by Que therapy independently of the presence/absence of diabetes (**Table 1**, EDV: treatment factor *p* < 0.05; ESV: treatment factor *p* < 0.01).

**Table 1:** Echocardiography data table of systolic and ventricular filling parameters.

	CI, ml/min/g	FS, %	EDV, ml	ESV, ml	HR, beats/min
**CQ pre-treatment**	0.110 ± 0.026	33.625 ± 0.632	1.231 ± 0.072	0.404 ± 0.031	309,500 ± 7,178
**DQ pre-treatment**	0.117 ± 0.014	33.933 ± 1.399	1.683 ± 0.103^Δ^	0.546 ± 0.048	280,467 ± 8,272
**CQ post-treatment**	0.105 ± 0.028	30.125 ± 1.209	1.534 ± 0.095	0.574 ± 0.040	316,857 ± 5,343
**DQ post-treatment**	0.102 ± 0.008	32.367 ± 0.834	1.844 ± 0.098	0.631 ± 0.038	290,000 ± 7,296
		* treatment factor	* treatment factor *** group factor	** treatment factor * group factor	** group factor

Cardiac index (CI) (calculated as the cardiac output divided by body weight), fractional shortening (FS), end-diastolic volume (EDV), end-systolic volume (ESV) and heart rate (HR) were analyzed in CQ and DQ group at two different time points – pre-treatment (week 0 – prior to the administration of Que) and post-treatment (week 6 – at the end of the Que treatment course). Data are presented as mean ± SEM and statistical differences as *p < 0.05, **p < 0.01, ***p < 0.001 treatment/group factor, ^Δ^ *p* < 0.05 vs. CQ pre-treatment (two-way ANOVA paired with Holm-Sidak´s multiple comparisons test).

### Quercetin reduced LV mass thickness and increased the internal diameter of LV

**Figure 2:**
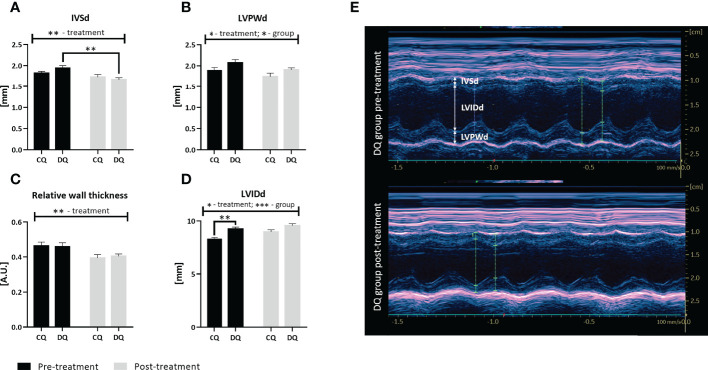
The assessment of LV structural parameters at two different time points – pre-treatment (week 0 – prior to the administration of Que) and post-treatment (week 6 – at the end of the Que treatment course). **(A)** Interventricular septal thickness in end-diastole (IVSd). **(B)** Left ventricular posterior wall thickness in end-diastole (LVPWd). **(C)** Relative wall thickness. **(D)** Left ventricular internal diameter in end-diastole (LVIDd). **(E)** Representative echocardiographic image of LV wall structure captured in Motion-mode in DQ group pre-treatment vs. post-treatment. Data are presented as mean ± SEM and statistical differences as *p < 0.05, **p < 0.01, ***p < 0.001 (two-way ANOVA paired with Holm-Sidak´s multiple comparisons test).

Interestingly, the echo analysis of LV wall structure, as displayed in **Figure 2**, revealed that Que was able to reduce both IVSd and LVPWd together with RWT (**Figure 2A**, IVSd: treatment factor *p* < 0.01; **Figure 2B**, LVPWd: treatment factor *p* < 0.05; **Figure 2C**, RWT: treatment factor *p* < 0.01), however, these effects were largely diabetes-independent. The LVPWd was also increased in diabetic animals (**Figure 2B**, group factor *p* < 0.05). Moreover, we detected a significant decrease in IVSd DQ post-treatment vs. DQ pre-treatment (**Figure 2A**, *p* < 0.01), highlighting the Que-mediated effects in diabetic animals. Overall, the 1-year-old animals did not show overt signs of LV hypertrophy with regards to the structural parameters. In contrast, Que potentiated increase and at the same time normalized the LVIDd (**Figure 2D**, treatment factor *p* < 0.05) which was significantly increased before the treatment in obese ZDF rats in comparison to lean controls (**Figure 2D**, DQ pre-treatment vs. CQ pre-treatment *p* < 0.01 and group factor *p* < 0.001).

### Quercetin attenuated pro-hypertrophic gene transcription-regulating HDAC4/MEF2 pathway and reduced LV collagen content

**Figure 3:**
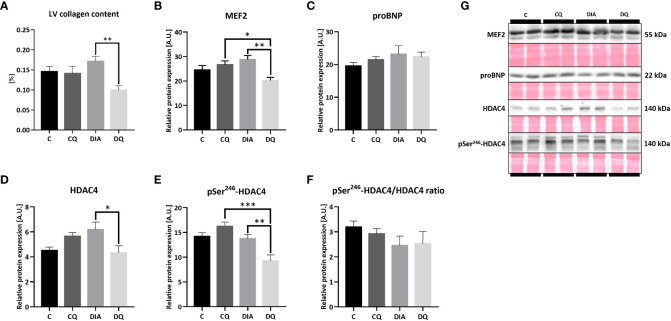
**(A)** LV total collagen content determined by hydroxyproline assay. **(B–F)** Western blot analysis of relative protein expression of **(B)** myocyte enhancer factor-2 (MEF2), **(C)** pro B-type natriuretic peptide (proBNP) **(D)** histone deacetylase 4 (HDAC4), **(E)** pSer^246^-HDAC4 and **(F)** pSer^246^-HDAC4/HDAC4 ratio. **(G)** Representative Western blots of detected proteins with estimated molecular weight (kDa) compared to total protein staining with Ponceau S. Data are presented as mean ± SEM and statistical differences as *p < 0.05, **p < 0.01, ***p < 0.001 (one-way ANOVA paired with Tukey´s multiple comparisons test).

As was previously described, diabetes affects the myocardium on multiple levels, causing myocardial stiffness, collagen deposition and exacerbated fibrosis (5). In terms of LV collagen accumulation, **Figure 3A** is showcasing the LV total collagen content which was selectively reduced in diabetic rats treated with Que when compared to vehicle-treated rats (DQ vs. DIA *p* < 0.01) without any observed effect on healthy control animals.

The immunoblotting analysis of the MEF2/HDAC4 transcriptional pathway regulating hypertrophic gene program (14) revealed a significant decrease in relative protein expression of MEF2 in the diabetic Que-treated group when compared to the diabetic vehicle-treated group (**Figure 3B**, DQ vs. DIA *p* < 0.01) as well as in comparison with control Que-treated group (**Figure 3B**, DQ vs. CQ *p* < 0.05), showing the ability of Que to inhibit MEF2 selectively in diabetic animals. Que behaved similarly regarding HDAC4, whose protein expression in diabetic animals was attenuated after Que treatment when compared to vehicle-treated diabetic rats (**Figure 3D**, DQ vs. DIA *p* < 0.05). The phosphorylation of HDAC4 at Ser^246^, which diminishes the inhibitory effects of HDAC4 upon MEF2 and promotes its export out of the nucleus (14), was similarly reduced as a result of Que treatment in diabetic group vs. vehicle-treated diabetic animals (**Figure 3E**, DQ vs. DIA *p* < 0.01) and in comparison to control Que-treated animals as well (**Figure 3E**, DQ vs. CQ *p* < 0.001). **Figure 3F** is showcasing normalized phosphorylated to total HDAC4 ratio. These observations are depicting the selective effects of Que to reduce pSer^246^-HDAC4 expression in Que-treated diabetic rats. The relative cardiac tissue expression of pro B-type natriuretic peptide (BNP), a significant marker of progressed heart failure, remained unaffected neither by diabetes or quercetin (**Figure 3C**). Representative Western blots are displayed in **Figure 3G**.

### The mitigation of pro-hypertrophic NFAT/calcineurin network as a result of Que therapy

**Figure 4:**
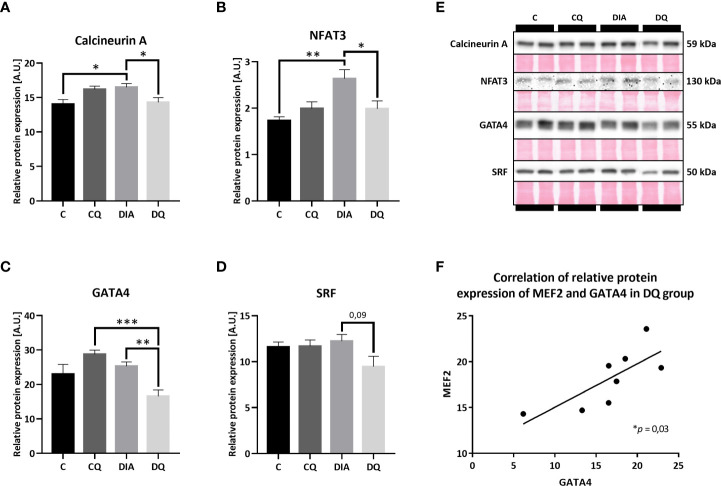
Western blot analysis of relative protein expression of **(A)** calcineurin A **(B)** nuclear factor of activated T cells 3 (NFAT3), **(C)** GATA4 transcription factor, **(D)** serum response factor (SRF). **(E)** Representative Western blots of detected proteins with estimated molecular weight (kDa) compared to total protein staining with Ponceau S. **(F)** The linear regression analysis of correlation between relative protein expression of MEF2 and GATA4 in DQ group. Data are presented as mean ± SEM and statistical differences as: *p < 0.05, **p < 0.01, ***p < 0.001 (one-way ANOVA paired with Tukey´s multiple comparisons test).

Being a part of the calcium-regulated mechanisms, the calcineurin/NFAT3 pathway is orchestrating multiple pro-hypertrophic genes in response to hypertrophic stimuli (34). In our model of T2DM, we observed significantly increased relative protein expression of both calcineurin A (**Figure 4A**, DIA vs. C *p* < 0.05) and NFAT3 (**Figure 4B**, DIA vs. C *p* < 0.01) in diabetic vehicle-treated animals compared to their lean controls. Simultaneously, Que treatment selectively attenuated overexpression of calcineurin A (**Figure 4A**, DQ vs. DIA *p* < 0.05) and NFAT3 (**Figure 4B**, DQ vs. DIA *p* < 0.05) in diabetic animals, while manifesting no effect on control lean animals. After obtaining the initial results, we further analyzed the expression profile of GATA4 and SRF factors. The level of GATA4, which is known to interact with and co-activate MEF2 (35), was significantly reduced by Que in diabetic rats in comparison with vehicle-treated diabetic animals (**Figure 4C**, DQ vs. DIA *p* < 0.01) and, in comparison with Que-treated lean animals (**Figure 4C**, DQ vs. CQ *p* < 0.001), indicating selective effects of Que on GATA4 inhibition in diabetic animals. Moreover, we found a significant correlation between GATA4 and MEF2 in the Que-treated diabetic group (**Figure 4F** *p* < 0.05). In the detected relative protein expression of SRF, there was a considerable trend (**Figure 4D**, DQ vs. DIA *p* = 0.09) towards decreased SRF in Que-treated diabetic animals vs. vehicle-treated diabetic animals. Representative Western blots are highlighted in **Figure 4E**.

### Upstream molecular pathways involved in anti-hypertrophic Que signalization

**Figure 5:**
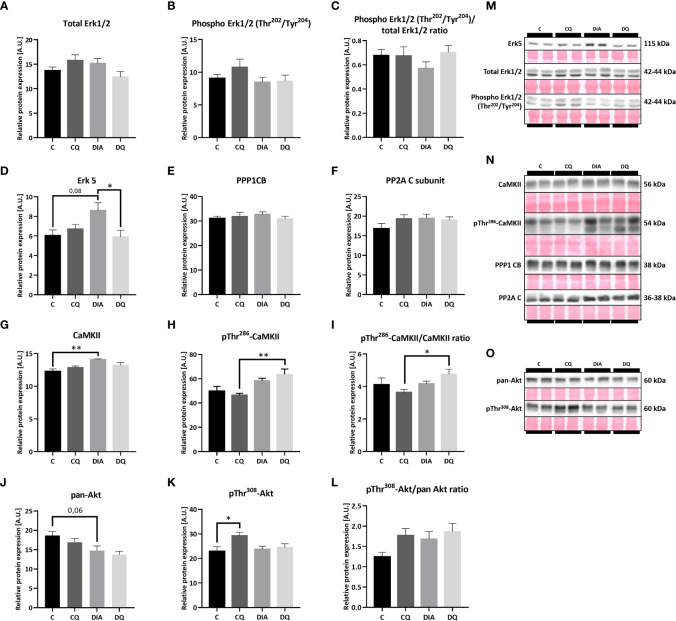
Western blot analysis of relative protein expression of **(A)** total extracellular signal-regulated kinase 1/2 (Erk1/2), **(B)** Phospho Erk1/2 (Thr^202^/Tyr^204^), **(C)** Phospho Erk1/2 (Thr^202^/Tyr^204^)/total Erk1/2 ratio **(D)** extracellular signal-regulated kinase 5 (Erk5), **(E)** Protein Phosphatase 1 Catalytic Subunit Beta (PPP1CB), **(F)** Protein Phosphatase 2A C Subunit (PP2A), **(G)** calcium/calmodulin-dependent protein kinase II (CaMKII), **(H)** pThr^286^-CaMKII, **(I)** pThr^286^-CaMKII/CaMKII ratio, **(J)** pan-Akt (protein kinase B), **(K)** pThr^308^-Akt, **(L)** pThr^308^-Akt/pan Akt ratio, **(M–O)** Representative Western blots of detected proteins with estimated molecular weight (kDa) compared to total protein staining with Ponceau S. Data are presented as mean ± SEM and statistical differences as: *p < 0.05, **p < 0.01 (one-way ANOVA paired with Tukey´s multiple comparisons test).

Subsequently, we examined multiple potential upstream pathways that might explain the observed effects of Que. Based on the fact that the transcriptional gene program can be activated by several cytosolic pathways (6) we screened the most prominent and most likely-activated pathways that are associated with cardiac remodeling. Firstly, we looked at MAPK family and found that Que significantly attenuated relative protein expression of Erk5 in the diabetic group when compared to vehicle-treated diabetic animals (**Figure 5D**, DQ vs. DIA *p* < 0.05). Moreover, we observed a trend toward increased Erk5 expression in association with diabetes (**Figure 5D**, DIA vs. C *p* = 0.08). This observation might render Erk5 as a possible upstream regulator of effects of Que in ZDF rats, as it was selectively modulated in diabetic animals while having no effect on lean controls. The expression of Erk1/2 as well as its phosphorylated form was maintained throughout the experiment (**Figures 5A–C**). Secondly, we investigated the calcium-dependent signalization pathway ruled by CaMKII which is activated by calcium-dependent autophosphorylation and on the other hand inhibited by dephosphorylation *via* protein phosphatases (36). However, neither CaMKII nor pThr^286^-CaMKII were affected by Que. We were only able to detect diabetes-induced changes, since diabetes increased the expression of both CaMKII (**Figure 5G**, DIA vs. C *p* < 0.01) and pThr^286^-CaMKII (**Figure 5H**, DQ vs. CQ *p* < 0.01), together normalized as pThr^286^-CaMKII/CaMKII ratio (**Figure 5I**, DQ vs. CQ *p* < 0.05) in LVs of ZDF rats. The levels of neither of the investigated protein phosphatases – PP1 or PP2A were altered (**Figures 5E, F**). Lastly, as shown in **Figures 5J–L**, the total Akt expression was unaffected by Que, we were only able to observe a trend towards decreased levels of Akt in diabetic animals compared to lean controls (**Figure 5J**, DIA vs. C *p* = 0.06). The Thr^308^ phosphorylated form of Akt was markedly increased in control animals treated with Que (**Figure 5K**, CQ vs. C *p* < 0.05) but was unchanged in diabetic group. Representative Western blots are displayed in **Figures 5M-O**.

## Discussion

Quercetin has been a compelling molecule in biomedical research for many years as a bioactive flavonoid that is well-tolerated and defined by a multifactorial spectrum of actions (21). Despite previous evidence demonstrating that Que possesses chemoprotective (37), neuroprotective (38) or cardioprotective effects (22–28), the therapeutical implications of Que in certain pathologies are limited by the lack of data on its cellular, protein or even nuclear targets. The exact molecular interactions are even vaguer in the model of cardiac disease triggered by diabetes mellitus. To gain more clarity, we designed a study that monitored Que-mediated cardiac effects in settings of T2DM accompanied by obesity. For this, we used an established model of ZDF rats harboring mutation in the leptin receptor gene.

Diabetes is known to provoke serious cardiac disbalance leading to cardiac remodeling (1, 4, 5) therefore we wanted to access the echocardiographic features of the heart as well as the proteomic profile. One of the key findings of this study was that prior to treatment, the obese diabetic animals had an increased E/A ratio compared to their lean controls. This increase was then selectively reversed by Que after the chronic 6-week-long treatment (**Figure 1**). Alteration of E/A ratio is used as one of the diagnostic markers for LVDD which can be oftentimes difficult to diagnose in clinical settings due to the asymptomatic nature of this condition (39). Previous studies similarly detected normalization of E/A ratio as a result of Que therapy (40–43), such as in Wistar rats fed with a high-cholesterol diet in which Que attenuated the reversal of E/A ratio (41). The E/A ratio dysregulation is a sensitive matter regarding diastolic function and highly depends on the underlying condition, both reversal and increase indicate an altered function, in our case, the increased E/A ratio seems to be leaning more toward the restrictive filling pattern (39). As the plasma triglycerides, cholesterol and LDL cholesterol of aged ZDFs remained unchanged by treatment, although raised by diabetes (31), it is less likely that the protective mechanisms by which Que contributes to the improvement of LVDD lies in the improvement of lipid metabolism. To bring more insight into the degree of present LVDD,we evaluated proBNP expression. Increase of inactive BNP underscores the severity of chronic heart failure (44) and can also indicate the level of diastolic dysfunction, since slight elevation of BNP might worsen the diastolic function in T2DM patients (45). In our study, tissue levels of proBNP (**Figure 3C**) remained unchanged which implies that the level of diastolic dysfunction in ZDFs was rather mild, similar results were previously obtained by Daniels et al. (46).

The systolic function of ZDF rats, evaluated according to the CI, remained stable (**Table 1**), while HR was decreased in diabetic groups, which is not uncommon in animals experiencing hyperglycemia (41, 47), and was not affected by Que treatment. Even though this study recruited aged 1 year old rats in contrast to more frequently used younger animals, these results were supported by similar studies on aged ZDFs which comparably detected signs of diastolic impairment with preserved or slightly impaired systolic function (46, 48). In a different model of streptozotocin-induced diabetes, reduced systolic function in terms of ejection fraction and FS was improved by Que (42). Contrastingly, in our model Que reduced the FS independently of diabetes and increased EDV and ESV (**Table 1**). Even though we do not consider these changes beneficial, they could play a part in the pseudo-normalization phase in cardiac adaptation to pathological stimuli. However, the effects linked to the LV structural parameters were far more interesting. Que was able to significantly reduce the thickness of LV mass, both IVSd and LVPWd and overall RWT and increased the internal diameter of LV in a diabetes-independent manner (**Figure 2**). We evaluate these effects as a sign of Que potency to act as a potential anti-hypertrophic agent, even though a more complex analysis is required in the model of combined obesity and T2DM. In line with our findings was a similar study of hypercholesterolemic rats in which Que likewise reduced the LV wall thickness and improved the LV luminal area but only in diseased rats (41). As T2DM is not only hypertrophy-provoking but also a fibrosis-provoking disease with documented increase of interstitial and perivascular fibrosis in hearts of ZDFs (49), we performed a follow-up analysis to assess LV collagen content. Que has previously manifested its protective actions against cardiac fibrosis and collagen accumulation (50, 51) which was in line with our findings that Que lowered total LV collagen in diabetic ZDF rats (**Figure 3A**).

**Figure 6:**
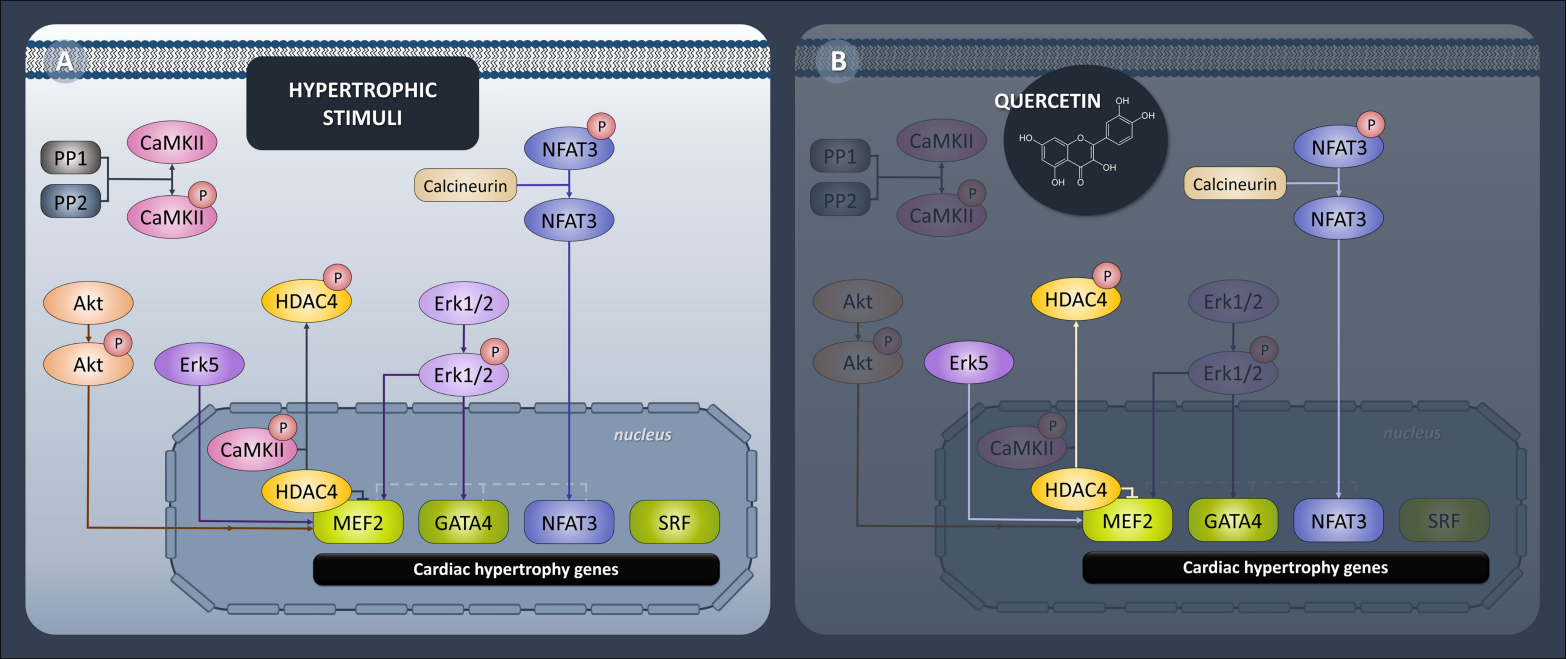
Diagrammatic representation of epigenetic and transcriptional control of hypertrophic signaling in heart. **(A)** Generally accepted protein pathways mediating cardiac hypertrophy. **(B)** Pathways targeted by quercetin in ZDF (Zucker Diabetic Fatty) rats. Quercetin attenuated expression of transcription factors MEF2, GATA4 and NFAT3 and downregulated upstream effectors HDAC4, calcineurin and Erk5.

All of the presented Que effects concerning protein expression profile were diabetes-selective which is the desired outcome presuming that Que can specifically target the diseased animals without affecting physiological values. One of the characteristics of diabetic myocardium is the presence of LVH which involves re-activation of fetal gene program promoting signal-dependent expression of structural proteins and hypertrophic growth (52). These analyses include novel findings, as Que effects have not been studied in association with protein pathways discussed below in a model of ZDF rats. In the current study, we found a connection between Que and pro-hypertrophic signalization in obese T2DM rats, as depicted in **Figure 6**. Since Que effectively penetrates the cellular and nuclear membranes and may accumulate in the cell nucleus as well as form protein and DNA bonds (53, 54), we hypothesized that Que might actively participate in the regulation of HDAC4 and respective pathways encompassing transcriptional factors mediating cardiac hypertrophy. We found that Que reduced MEF2 expression together with HDAC4 and pSer^246^-HDAC4 in diabetic animals (**Figure 3**). When active, MEF2 functions to promote physiological heart development or hypertrophic growth as an adaptation to stress/pathological stimuli. HDAC4 on the contrary inhibits these actions of MEF2 until it becomes phosphorylated which triggers the nuclear export of HDAC4 while derepressing its inhibitory properties upon MEF2 (14). Decreased phosphorylation of HDAC4 at Ser^246^ could indicate that fewer molecules of HDAC4 are exported out of the nucleus and are available to repress MEF2-dependent transcription in the nucleus. The halting of MEF2 activity is known to attenuate the adverse effects in cardiac remodeling (11) and also in the diabetic myocardium (7–9, 55). Similarly, HDAC4 inhibition proved to be beneficial against the development of diabetic cardiomyopathy (56). Que was also found to inhibit HDACs dose-dependently and selectively reduce the activity of HDAC4 in bovine heart tissues (57). Our observations together with the previous accounts support the role of Que in the cardiac epigenetic modulation of nucleosome landscape and advocate for its cardioprotective actions.

The effects of Que go even further, as we detected reduced GATA4 and NFAT3 expression in treated diabetic animals (**Figure 4**). Both are MEF2 co-activators as well as important mediators in the cardiac hypertrophy (15, 16). However, their precise role in the regulation of diabetes-induced LVH is still poorly understood. Elevated GATA4 expression was previously associated with diabetes-provoked adverse effects and was prevented by N-acetyl cysteine treatment (17). Furthermore, in a model of cardiac hypertrophy, Que was found to prevent a hypertrophy-induced increase of GATA4 (43). In the current model, we were able to link Que with the attenuation of GATA4 in diabetic myocardium. NFAT3 was on top of that significantly induced in diabetic ZDF rats and this was prevented by Que. The same pattern was detected for the expression of calcineurin A (**Figure 4A**), a protein phosphatase that essentially dephosphorylates NFAT3 allowing its nuclear import and activating its transcriptional properties (15). Previous reports associated enhanced NFAT3/calcineurin pathway with LVH (58) also under hyperglycemic stress (59) and reduced NFAT3/calcineurin with amelioration of LVH which is in agreement with our results.

The next step was to screen for a potential upstream signaling cascade of analyzed transcriptional factors. Even though we ruled out multiple signaling pathways like the calcium-dependent CaMKII pathway or involvement of protein phosphatases, Erk1/2 or Akt kinase, we discovered that Erk5 expression was enhanced in diabetic animals and considerably attenuated in Que-treated diabetic animals (**Figure 5D**). Erk5 is an essential upstream integrator of multiple cellular processes and is expected to be a part of the cardiac hypertrophic response that is associated with the MEF2 activity (20). Studies concerning Erk5 role in diabetic/obese myocardium recorded various results of Erk5 expression in the hearts of diseased animals (60), however when we look at studies researching Erk5 as an upstream for hypertrophic growth, the attenuation of its activity seems to be beneficial (20). Que has so far been linked to Erk5 by a recent study of aortic aneurysm and dissection in mice, which reported that Que activated Erk5. Under those settings, Erk5 phosphorylation might lead to endothelial nitric oxide synthase expression and contribute to endothelial protection (61).

In this study, we have demonstrated for the first time that Que was able to mitigate developed diastolic dysfunction and did it selectively in diabetic ZDF rats. Moreover, Que promoted an overall reduction of LV wall thickness while increasing the luminal area. With regards to the proteomic pathways mediating LVH in diabetic rats, Que was linked to the amelioration of pro-hypertrophic signaling through the modulation of MEF2/HDAC4, calcineurin/NFAT and associated pathways. The main outcome of the current study is the novel link between Que and analyzed proteomic pathways in diabetic myocardium of ZDF rats which might contribute to its cardioprotective effects. However, further exploration is still needed to clarify the proposed mechanisms.

## Data availability statement

The raw data supporting the conclusions of this article will be made available by the authors, without undue reservation.

## Ethics statement

The animal study was reviewed and approved by Animal Research and Care Committee of the Centre of Experimental Medicine SAS—Project no. 2237/18-221/3.

## Author contributions

Supervision of the study: MB and TR. Experimental design: KF, BK, MB, and TR. Data collection: LB, CH, PG, KF, BK, AS, and TR. Statistical analysis: LB and TR. Manuscript drafting: LB. Manuscript revision: LB, CH, PG, KF, BK, AS, AD-A, MB, and TR. All authors have revised and approved the submitted version of the article.
